# The Developing Human Sphenoid Bone: Linking Embryological Development to Adult Morphology

**DOI:** 10.3390/biology14081090

**Published:** 2025-08-20

**Authors:** George Triantafyllou, Maria Piagkou

**Affiliations:** 1 Department of Anatomy, School of Medicine, Faculty of Health Sciences, National and Kapodistrian University of Athens, 11527 Athens, Greece; georgerose406@gmail.com; 2 “VARIANTIS” Research Laboratory, Department of Clinical Anatomy, Masovian Academy in Płock, 09400 Płock, Poland

**Keywords:** sphenoid bone, embryology, anatomy, variation, ligaments, foramina, developmental anatomy

## Abstract

The human sphenoid bone (SB), centrally located at the cranial base, is structurally and developmentally complex. It arises from multiple cartilaginous precursors and undergoes both endochondral and intramembranous ossification, forming essential elements such as the sella, orbital walls, and numerous foramina. This review integrates embryological, anatomical, and radiological findings to present a comprehensive view of SB development and variation. Embryological studies reveal a layered ossification sequence, with accessory centers in the presphenoid and basisphenoid that influence adult morphology and variants, such as the caroticoclinoid foramen. In adulthood, the SB consists of a central body, paired greater and lesser wings, and the pterygoid processes, which articulate with key craniofacial bones and transmit vital neurovascular structures. Notable variants include duplication or absence of foramina, ossification of ligaments such as the pterygoid and pterygospinous ligaments, and the formation of bony bridges among the clinoid processes. These variants may affect cranial nerve trajectories and surgical access, posing potential risks during neurosurgical, endoscopic, and dental interventions. Emissary structures such as the sphenoidal emissary foramen and the newly described sphenopterygoid canal underscore the region’s vascular complexity. Additionally, variations in the optic and Vidian canals, as well as the superior orbital fissure, can also impact surgical approaches to the orbit, sinuses, and skull base. Understanding the full spectrum of sphenoid bone embryogenesis and morphology is essential for safe clinical practice and practical radiological imaging.

## Simple Summary

The sphenoid bone is a centrally located bone at the base of the human skull. It is crucial for supporting the brain, forming parts of the eye socket, and allowing passage for important nerves and blood vessels. This bone develops in a complex way before birth, combining cartilage-based and membrane-based processes. Its formation is closely tied to the development of the pituitary gland and other parts of the brain. As the sphenoid matures, it develops many holes (foramina) and bony structures that can vary between individuals. These variations can affect surgical access and increase the risk of complications during procedures near the skull base. Understanding how this bone forms and changes can help improve diagnosis, surgery, and treatment of related conditions.

## 1. Introduction

Embryology and anatomy are intrinsically linked disciplines within human morphology. The embryological development of the human skull remains a complex and only partially understood process. Numerous studies continue to explore how the skull acquires its mature form. This structure is not only morphologically intricate but also functionally critical, as it houses the brain and special sensory organs and provides passage for essential neurovascular structures. Recent research utilizing histological sections of human fetuses has significantly advanced our understanding of cranial development [1,2,3,4]. These studies have significantly enhanced our understanding of cranial base development, providing crucial insights into the sequential ossification processes and the intricate relationships between cartilaginous precursors and the final bony structures.

This growing body of research continues to illuminate the developmental trajectory of key bones, including the frontal, occipital, ethmoid, and sphenoid bone (SB), as well as the paired temporal and parietal bones. Each of these bones exhibits distinct anatomical features and foramina that contribute to the formation of the cranial cavity and the safe passage of neurovascular elements. Among these, the SB is particularly notable for its structural complexity and central position within the cranial base. The SB connects with numerous other cranial bones and houses critical neurovascular pathways [5]. The SB’s embryological development involves a unique combination of endochondral and intramembranous ossification, influenced by both neural crest-derived mesenchyme and chondrogenic structures, which ultimately lead to its distinctive shape and form [6].

This dual process is essential in forming key anatomical features, such as the greater and lesser wings (GWs and LWs), and the basisphenoid, which form the bone’s complex structure. While numerous studies have examined its embryological origins, investigations into its adult morphology often focus on the formation and variations of foramina and ligamentous structures, such as the optic canal (OC), foramen ovale (FO), and pterygoid processes (PPs), which facilitate the passage of cranial nerves and blood vessels [5].

This scoping review aims to integrate current embryological and anatomical findings, establish connections between developmental processes and adult morphologies, and highlight their relevance to clinical and surgical practice. By bridging the gap between embryological development and the morphological variants observed in adulthood, this work emphasizes the clinical significance of SB morphology in various surgical approaches to the skull base, particularly in neurosurgery, endoscopic procedures, and maxillofacial surgery.

## 2. Embryological Development of the Human Sphenoid Bone

**Figure 1:**
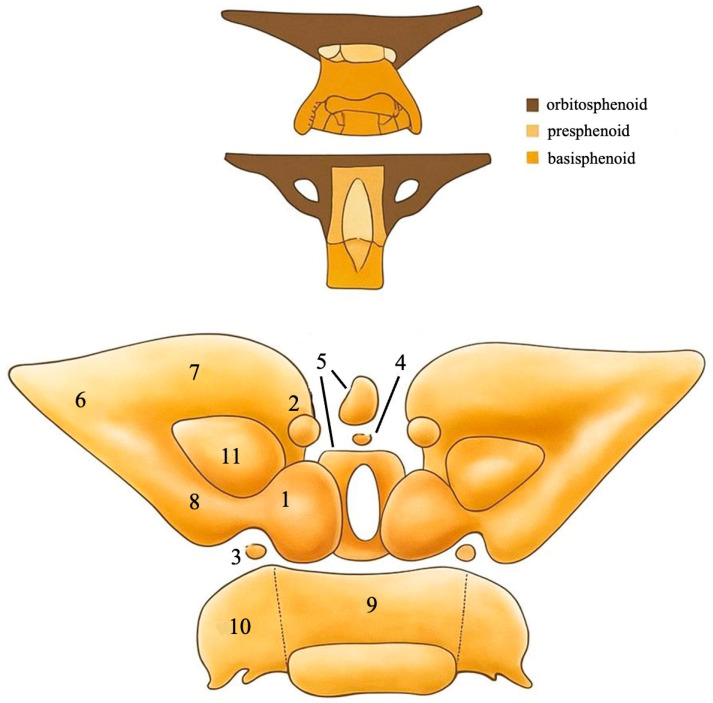
An anterior view of the ossification centers of the presphenoid and the other parts of the sphenoid bone. (1) Main primary center of the presphenoid, (2) anterior accessory center of the presphenoid, (3) posterior accessory center of the presphenoid, (4) middle accessory center of the presphenoid, (5) middle body ossification center of the presphenoid, (6) orbitosphenoid, (7) anterior part of the orbitosphenoid, (8) posterior part of the orbitosphenoid, (9) middle basisphenoid, (10) lateral basisphenoid, (11) optic canal [3,4,5,6,7,8,9,10]. Adapted from [3,10].

The SB, centrally located at the base of the skull, develops through a highly coordinated process involving both endochondral and intramembranous ossification. It arises from multiple cartilaginous precursors, including the basisphenoid, orbitosphenoid, ala temporalis, and alar process. This embryogenesis reflects complex interactions between chondrogenic structures, neural crest-derived mesenchyme, brain expansion, and cranial nerve trajectory [3,6,7,8]. Recent advancements in imaging and histological techniques have provided a more detailed understanding of how these structures interrelate during early development [9,10] (Figure 1).

The SB develops from multiple embryonic cartilaginous elements that originate in the cranial base during early gestation [3,4,5,6,7,8,9,10]. These components include

- Presphenoid: Contributes to the anterior body of the SB and forms the floor of the anterior cranial fossa.
- Basisphenoid: Forms the posterior body of the SB, including the sella turcica and clivus region.
- Orbitosphenoid: Gives rise to the LWs, forming part of the anterior cranial fossa and the roof of the orbit.
- Alisphenoid: Forms the GWs, contributing to the middle cranial fossa, the lateral orbital wall, and the infratemporal fossa.
- Ala temporalis and alar process: Transient cartilaginous structures that serve as key intermediates in the development of the GWs and PPs.

Each of these elements arises from distinct ossification centers and later fuses to form the mature, complex architecture of the adult sphenoid [3,4,5,6,7,8,9,10].

Historically, Sutton [7] offered one of the earliest detailed accounts of SB development, describing the fusion of the basisphenoid and ligulae ossification centers, as well as the initial ossification within the ala (alisphenoid), orbitosphenoid, and presphenoid regions. Sutton’s pioneering work laid the groundwork for understanding the foundational elements of the SB’s embryonic formation, particularly the complex interplay between cartilaginous and membranous elements that form the adult bone. He also noted the temporary persistence of the craniopharyngeal canal, a midline epithelial-lined passage traversing the basisphenoid during mid-gestational development. Sutton drew a developmental analogy between this canal and the neurenteric canal, situating the former at the rostral end and the latter at the caudal end of the spinal axis [7]. The craniopharyngeal canal is closely associated with Rathke’s pouch, the ectodermal precursor to the anterior pituitary. Disruptions in the development or involution of Rathke’s pouch and associated midline structures can lead to anomalies such as craniopharyngiomas, as previously described by Chun et al. [11]. These insights underscore the embryological relevance of Sutton’s observations, particularly in the morphogenesis of the hypophyseal fossa and sellar region.

Recent studies have provided a more refined understanding of these early developmental stages, illustrating how the trabecular system forms a supportive framework for subsequent ossification centers, eventually contributing to the adult SB’s intricate structure [9,11]. Recent insights into the neural crest-derived mesenchyme have further clarified the role of neuroectodermal tissue in the formation of sphenoidal precursors. For example, Yamamoto et al. [6] and Hirouchi et al. [3] explored how neural crest-derived cells contribute to the lateral development of the SB, particularly in the formation of the GWs and LWs, as well as the PPs. These studies highlight the crucial role of neural crest cells in orchestrating the early morphogenetic movements that shape the adult form of the SB [6]. Further studies, such as those by Grzonkowska et al. [12] and Zhang et al. [13], have provided detailed descriptions of how the SB ossifies in tandem with surrounding structures, thereby contributing to the formation of key anatomical landmarks, including the sella and OC. Grzonkowska et al. [12] also noted the simultaneous development of adjacent bones, such as the occipital bone, and how their integration with the SB forms the complete cranial base architecture. This highlights the interconnectedness of the skull’s embryonic development.

### 2.1. Refinement of Ossification Patterns and Presphenoid Complexity

Kodama’s [14] meticulous analysis of 70 fetal specimens revealed that the presphenoid region exhibits greater complexity in its ossification than previously acknowledged. Kodama identified five distinct types of ossification centers associated with the presphenoid:

1. Main center—the earliest to appear (as early as ~17 cm craniocaudal length- CRL, ~8–9 weeks gestation).
2. Anterior accessory centers—emerge around 24–25 cm CRL (~10–11 weeks), and by late fetal stages are consistently observed.
3. Posterior accessory centers—rarely appear before 31 cm CRL and often remain incompletely ossified during gestation.
4. Middle accessory center—appears in later stages (after ~34 cm CRL), suggesting a late and possibly incomplete ossification process.
5. Corporal middle centers—deep-layered ossific centers that become evident only after ~34 cm CRL and show increasing presence into the 9th and 10th months of gestation

These findings support a layered and sequential model of ossification, wherein superficial centers develop before deeper, more central ones. The progressive fusion of these centers—linking the presphenoid with the basisphenoid, orbitosphenoid, and contralateral presphenoid—advances throughout the third trimester [14]. This model illustrates how the initial cartilaginous elements give rise to the complex structure of the adult sphenoid, enabling the differentiation of key features, such as the sella and the GWs and LWs [14].

Further reinforcing this developmental model, Zdilla [15] investigated the formation of the caroticoclinoid foramen (CCF) in fetal dried skulls and observed that it may arise as a subtle bony extension from the anterior and/or middle clinoid processes (ACPs and/or MCPs). He proposed that this process is potentially influenced by the presence or asymmetrical development of accessory presphenoid ossification centers, as outlined by Kodama, leading to variations in middle clinoid prominence and eventual formation of the CCF. This developmental insight aligns with the broader understanding that variations in ossification are crucial to understanding anomalies of the sphenoid, such as the CCF [15].

The role of these accessory centers in shaping the adult SB cannot be overstated, as they are responsible for key structural elements, particularly the formation of bony bridges and foramina that affect neurovascular pathways [11] (Table 1).

The key findings that should be noted from this section are

- The presphenoid exhibits a layered ossification model, with five distinct centers appearing sequentially.
- These centers contribute to the formation of the sella turcica, clinoid processes, and bony foramina, explaining common adult anatomical variants such as the CCF.

### 2.2. Initial Developmental Stages (6–8 Weeks of Development)

The formation of SB begins around the third week of gestation, under the influence of the notochord and surrounding mesenchymal tissues [16]. By approximately day 40, distinct cartilaginous primordia—namely the orbitosphenoid, alisphenoid, presphenoid, and postsphenoid—develop from separate centers of chondrogenesis [16]. These early precursors give rise to key components of the adult SB: the orbitosphenoid forms the LWs, the alisphenoid develops into the GWs, and the presphenoid and postsphenoid contribute to the anterior and posterior parts of the SB, respectively. These developing elements are initially separated by transient synchondroses, such as the intersphenoidal and spheno-occipital synchondroses, which regulate the segmented nature of early sphenoid ossification [16]. These synchondroses allow for the flexibility and growth of the sphenoid, facilitating its integration with other cranial structures during fetal development [11]. Moreover, by the sixth to seventh week of gestation, the notochord is observed extending beneath the dorsum sellae, playing a crucial inductive role in the formation of the posterior sphenoid body. The anterior wall of the sella forms in relation to Rathke’s pouch, the precursor of the anterior pituitary, which invaginates from the oral ectoderm. These interacting tissues contribute to the shaping of the hypophyseal fossa, a central feature of the developing cranial base and a key anatomical hallmark of the sphenoid bone [12,15].

At Carnegie stage 16 (~6 weeks gestation), these cartilaginous structures first appear as undifferentiated mesenchymal condensations. The initial fusion of these mesenchymal elements plays a crucial role in the early morphogenesis of the SB, with individual ossification centers gradually becoming more defined [16]. The ala temporalis, alar process, and associated cranial ganglia are visualized as a single mass at this stage [8]. The basisphenoid sits centrally, bordered anteriorly by the orbitosphenoid and laterally by the ala temporalis. A key morphological feature—the alar process—links the basisphenoid to the ala temporalis, creating a distinctive “key-and-keyhole” configuration. This structural arrangement plays a stabilizing role during early morphogenesis of the sphenoidal cartilage, with the alar process contributing to the structural integrity of the cranial base [6]. This configuration is crucial for maintaining the overall alignment of the developing sphenoid, thereby ensuring proper spatial relationships between adjacent cranial structures [3,6].

The key findings that should be noted from this section are

- The orbitosphenoid, alisphenoid, presphenoid, and postsphenoid develop as independent cartilaginous elements.
- Transient synchondroses (e.g., intersphenoidal) and structures like the alar process stabilize early sphenoid morphogenesis.

### 2.3. Ossification Dynamics and Morphological Growth (8–30 Weeks of Gestation)

By 8–10 weeks of gestation, the cartilaginous primordia of the SB begin to undergo endochondral ossification. The GWs and lateral pterygoid plates (LPPs), both derivatives of the alisphenoid, form through a combination of endochondral and intramembranous ossification [12]. Bone staining and computed tomography (CT) imaging studies have demonstrated early ossification foci at the LWs and GWs, as well as within the sellar region [13]. The orbitosphenoid contributes to the formation of the LW, while the ala temporalis gives rise to the GW. The alar process, however, remains cartilaginous and functions as a structural bridge between the basiocciput and the ala temporalis, serving as a key stabilizing element during early sphenoid development.

Membranous ossification also initiates near the ala temporalis, contributing to the formation of the PPs and surrounding foramina, including the foramen rotundum (FR) and foramen ovale (FO) [11]. These foramina, critical for the passage of cranial nerves and blood vessels, begin their formation during this phase, underscoring the importance of the SB as a conduit for neurovascular structures. Quantitative analyses have revealed distinct growth trajectories for the presphenoid and postsphenoid ossification centers. The presphenoid exhibits proportional growth in both sagittal length and volume, while the postsphenoid shows logarithmic increases in most linear dimensions, accompanied by proportional volumetric expansion [12]. This suggests that the presphenoid primarily contributes to the elongation of the body of the SB, while the postsphenoid plays a larger role in overall volumetric development. Utsunomiya et al. [17] applied 3D histological reconstruction to embryonic and fetal specimens, revealing dynamic shifts in chondrification and ossification at the cranial base. Their findings confirmed that the presphenoid and postsphenoid regions emerge as distinct cartilaginous structures with region-specific timing and vascularization patterns. Importantly, the timing of ossification center appearance aligned closely with neurovascular development, reinforcing the view that foramina form around pre-existing nerve pathways.

Further insights by Li et al. [10] mapped the spatial relationships among the frontal bone, ala minor, and cartilaginous ethmoid in the fetal orbital roof. Their findings demonstrated that the frontal bone consistently overlaps the ala minor and the superolateral portion of the ethmoid plate. Notably, ossification of the ala minor proceeds via endochondral mechanisms but lacks a typical hypertrophic cartilage zone, suggesting a simplified ossification front that contrasts with more complex ossification patterns seen in other cranial regions. This simplified ossification front is particularly significant as it influences the shape and growth of the fetal orbital roof and ultimately contributes to cranial stability during late gestation [10]. Between weeks 10 and 16 of gestation, regions associated with the ala temporalis, particularly those involved in forming the greater wing, begin to exhibit membranous ossification in adjacent mesenchymal condensations, while the core structure retains its cartilaginous framework. This reflects a dynamic regional shift in ossification mode, contributing to the final morphology of the ala major [10]. Based on the aforementioned studies [10,11,12,13,14,15], the SB’s formation involves three distinct developmental mechanisms:

- Endochondral ossification occurs in cartilage preformed regions such as the basisphenoid, presphenoid, orbitosphenoid, and alisphenoid, where cartilage is gradually replaced by bone.
- Intramembranous ossification is seen in areas derived from ectomesenchyme (neural crest-derived mesenchyme), particularly in the lateral regions of the GWs, PPs, and nearby facial bones, where bone forms directly within mesenchymal condensations without a cartilage intermediary.
- Periosteal appositional growth contributes to bone thickening and remodeling, especially at the junctions of ossification centers and during later fetal stages, refining the external contour and internal architecture of the SB.

These overlapping processes result in the complex and variable morphology of the mature sphenoid, making it one of the most developmentally intricate bones in the human skull.

### 2.4. Structural Integration and Maturation (15–34 Weeks and Beyond)

By the mid-fetal period (15–20 weeks of gestation), notable morphological variations in the SB begin to emerge. Zhang et al. [13] documented both single- and double-center ossification patterns in the basisphenoid, which influence the relative widths of the basisphenoid and orbitosphenoid, thereby impacting the overall configuration of the cranial base. These variations in ossification patterns are integral to the final shape of the SB and its relationship with adjacent structures such as the temporal and basioccipital bones. As these bones begin to ossify together, they facilitate the formation of crucial synchondroses such as the spheno-occipital synchondrosis, a vital growth site that typically fuses during adolescence [16]. Synchondrosis plays a crucial role in the flexibility of the developing cranial base, enabling continued growth while maintaining stability. Concurrently, the SB begins to ossify in conjunction with adjacent bones, such as the basiocciput and temporal bones, ensuring coordinated cranial development. This ossification is essential for establishing the adult configuration of the cranial base and its functional components [10].

The GW (ala major) also plays a transient role in orbital formation during fetal development. Hirouchi et al. [3] noted that GW initially contributes to the orbital structure before shifting medially as the maxilla and zygomatic bones grow and remodel the region. This dynamic process reflects the SB’s role not only as a structural anchor but also in the development of surrounding anatomical features, particularly in the formation of the orbit and zygomatic arch.

In the later stages of gestation, ossification centers expand, and surrounding membranous bones continue to develop, refining the contours of the adult SB morphology [11]. This continued development leads to a more integrated and mature sphenoid structure, where key components such as the dorsum sellae begin to project superiorly from the basisphenoid. In contrast, the sphenoid sinus initiates its formation from epithelial outpouchings of the nasal cavity. This process of sinus formation is crucial for the later development of the sphenoid’s aeration and its relationship with adjacent sinuses [11].

Additionally, interspecies variation in the development of the alar process has been observed. The presence or absence of this structure may cause anatomical differences, such as the formation of the pterygospinous bar (PTSB), suggesting that this variant may originate from different sphenoidal precursors across species [9,18]. These variations have significant implications for understanding the evolutionary and functional anatomy of SB, particularly in species with distinct cranial and facial morphology.

The key findings that should be noted from this section are as follows:

- Variations in basisphenoid and orbitosphenoid ossification influence final cranial base configuration.
- The spheno-occipital synchondrosis begins forming and will later act as a critical growth site into adolescence.

### 2.5. Formation of Associated Structures and Ligamentous Attachments

As ossification proceeds, foramina such as the FO and foramen spinosum (FS) form by the fusion of bony processes around nerves and vessels. The ossification of the posterior sphenoid, particularly GW, plays a crucial role in delineating these structures [19]. The GW of the SB not only contributes to the cranial base’s structural integrity but also ensures the precise spatial arrangement of critical neurovascular passages, such as the trigeminal nerve branches (V2 and V3), which traverse the FR and FO. The sphenoid spine, which emerges near the FS, completes formation late in fetal development and varies among individuals and species. This variability has implications for the mechanical and neurovascular stability of the cranial base during early development [19]. These foramina are phylogenetically relevant, and their ossification patterns display progressive medial ossification through human evolution, further supporting the theory that cranial base architecture is shaped by evolutionary processes [19].

Meckel’s cartilage, which extends from the developing mandible to the middle ear, plays a key role in the formation of the sphenomandibular ligament (SML) [20]. By 12 weeks, Meckel’s cartilage is seen inserting into the lingula of the mandible, with the surrounding connective tissue guiding the topography of the future ligament [20]. The sphenoid spine, a key landmark for the attachment of the SML, appears by 17 weeks as a center of intramembranous ossification dorsal to the middle meningeal artery [20]. This ossification not only contributes to the structural integrity of the SML attachment but also plays a role in the overall biomechanical properties of the developing cranial base.

Recent high-resolution histological reconstructions by Jin et al. [21] reveal that the posterior portion of Meckel’s cartilage undergoes segmentation into ligamentous and bony derivatives between 10 and 14 weeks of development. Notably, the authors describe a dual derivative of the forming SML, with one end extending anteriorly into the mandibular lingula and the other posteriorly into the perichondral tissue adjacent to the sphenoid spine and tympanic cavity [21]. This posterior condensation co-develops with ossification centers in the alisphenoid, highlighting the dynamic interplay between ligament formation and ossification. The study suggests that the migration of neural and vascular elements, including branches of the mandibular nerve (MN), is closely coordinated with the formation of these ligamentous structures [21]. Jin et al. (2024) emphasize that the ligament’s trajectory becomes clearer as surrounding mesenchymal tissues densify and collagen fibers align under tension, indicating that mechanical forces help shape its final form. Additionally, their reconstructions demonstrate that the ossification of the sphenoid spine not only serves as a structural anchor for the ligament but also aligns with the emergence of the FS, indicating a coordinated development of neural, vascular, and ligamentous components [21]. This integrated model supports the idea that cranial base architecture, including minor landmarks such as the sphenoid spine, is not only a result of ossification centers but also involves biomechanical and neurovascular interactions within the developing craniofacial complex.

The key findings that should be noted from this section are as follows:

- Foramina form around nerves and vessels; sphenoid spine develops as a key anchor for ligaments.
- Meckel’s cartilage contributes to SML formation, with neural and vascular elements guiding its trajectory.

### 2.6. Developmental Relationship with Neurovascular Structures

The role of the SB in shaping the course of the internal carotid artery (ICA) and the carotid canal has been clarified by Honkura et al. [22]. Between 10 and 18 weeks of gestation, the ala temporalis, pterygoid, and alar process of the sphenoid flank the ICA as it courses between the sphenoid and temporal bones. Notably, the carotid canal does not initially form as a discrete foramen. Instead, it begins as a loose mesenchymal space, which is gradually bordered by both cartilaginous and ossifying elements of the sphenoid [22]. This process marks a significant early phase in the development of neurovascular pathways, where the mesenchymal space serves as a precursor to the bony structures that will eventually form the carotid canal. After 25 weeks, the pterygoid process grows and elevates the pharyngotympanic tube, deflecting the ICA superiorly into a groove that gradually ossifies, forming part of the petrosal contribution to the canal [22]. The carotid canal wall is formed mainly through membranous ossification, primarily from mesenchymal tissue between the petrosa and the sphenoid, rather than from the sphenoid alone [22]. This discovery adds more nuance to the traditional view, which posited that the carotid canal is entirely petrosal in origin and highlights the sphenoid’s indirect yet essential role in shaping the neurovascular corridors of the skull base.

Li et al. [18] studied the ossification of nerve canals passing through the SB and surrounding bones. Their data confirm that the maxillary nerve, which travels through the ala temporalis and nearby sphenoidal cartilage, is encased by perichondral ossification. This pattern of ossification differs from that of membranous bones, where periosteal bone plays a significant role in ossification. These findings underscore the importance of biomechanical and neuroanatomical factors in shaping ossification pathways within the sphenoid complex, particularly in forming neurovascular channels that support critical structures, such as the maxillary nerve [18].

The key findings that it should be noted from this section are as follows:

- The ICA path is shaped by surrounding sphenoid and petrosal ossification.
- Perichondral ossification around cranial nerves (e.g., maxillary nerve) facilitates canal and foramen formation, establishing adult neurovascular corridors.

## 3. Traditional Anatomy of the Human Adult Sphenoid Bone

The adult human SB is a single, centrally located part of the cranial base, notable for its complex structure and key anatomical connections. It acts as a crucial element in cranial architecture, linking various regions of the skull. The SB contributes to the formation of several important areas, including the anterior and middle cranial fossae, the orbit, and the nasal cavity, as well as nearby fossae such as the temporal, infratemporal, and pterygopalatine fossae [23]. Shaped roughly like a bat or moth in flight, the sphenoid’s intricate form highlights its vital role in providing structural support and organizing the cranial cavity [5]. Anatomically, the SB connects with twelve bones, including the frontal, parietal, temporal, occipital, ethmoid, vomer, zygomatic, palatine, and maxillary bones. It consists of a central body and three pairs of processes: the GWs, LWs, and PPs [5]. The body contains the paired sphenoidal sinuses, separated by a thin septum and opening into the nasal cavity through the sphenoethmoidal recess [5]. This sinus system is essential for air circulation and maintains overall cranial pressure.

The GWs of the SB extend laterally and help form the middle cranial fossa, the lateral skull wall, and part of the orbit. They also allow passage for important structures such as the FR, FO, and FS, all of which are vital for neurovascular transmission [5]. The LWs, on the other hand, help form the anterior cranial fossa and the roof of the orbit, while also housing the OC, which carries the optic nerve (cranial nerve- CN II) and the ophthalmic artery. The PPs that descend from the junction of the body and GWs are divided into medial and lateral plates. The PPs, specifically the lateral pterygoid plate, serve as the origin for important muscles of mastication (the medial and lateral pterygoid muscles) and help define the boundaries of the pterygopalatine fossa [5].

### 3.1. The Sphenoidal Body

**Figure 2:**
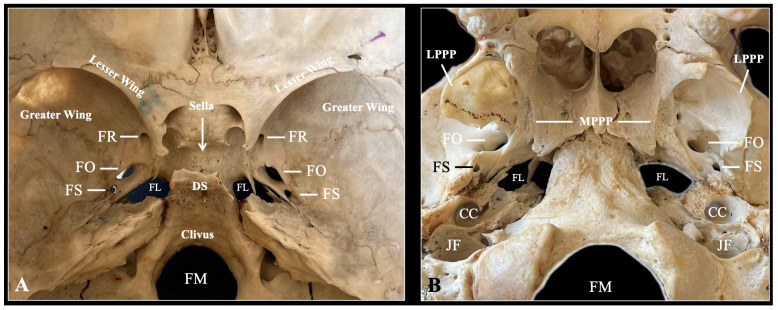
Typical anatomy of the sphenoid bone depicted through osteological specimen intracranial view (**A**) and extracranial view (**B**). FR—foramen rotundum; FO—foramen ovale; FS—foramen spinosum; FL—foramen lacerum; DS—dorsum sellae; FM—foramen magnum; LPPP—lateral pterygoid process plate; MPPP—medial pterygoid process plate; CC—carotid canal; JF—jugular foramen.

The cuboidal body of the SB contains the paired sphenoidal sinuses, separated by a thin bony septum [5]. On its superior (cerebral) surface, there is the jugum sphenoidale, which is continuous anteriorly with the cribriform plate of the ethmoid bone and bordered posteriorly by the tuberculum sellae, the sella, and the dorsum sellae. The pituitary gland resides within the hypophyseal fossa of the sella, flanked by the MCP and posterior clinoid processes (PCPs). These serve as attachment points for the diaphragma sellae and the tentorium cerebelli, respectively [5]. Lateral to the sella, the carotid sulcus accommodates the cavernous segment of the internal carotid artery (ICA), along with the abducens nerve (CN VI), which passes through the lumen of the cavernous sinus. In contrast, cranial nerves III (oculomotor), IV (trochlear), V1 (ophthalmic), and V2 (maxillary) are embedded within the lateral wall of the sinus [5]. An often overlooked but clinically important structure related to the sphenoid bone is Dorello’s canal—an osteofibrous channel at the base of the posterior clinoid process, bounded superiorly by the Gruber’s ligament. This canal transmits the abducens nerve (CN VI), the inferior petrosal sinus, and small arterial branches such as those from the meningohypophyseal trunk. The posterior clinoid’s spatial relationship with the petrous apex and dorsum sellae defines the canal’s roof and inferolateral wall, making it partly dependent on the morphology of the sphenoid bone [5]. Anteriorly, the SB contributes to the posterior roof of the nasal cavity and the nasal septum via the sphenoidal crest and rostrum. Inferiorly, the vaginal processes extend medially from the bases of the medial pterygoid plates, articulating with the vomer and palatine bones and participating in the formation of the palatovaginal canal [5] (Figure 2).

### 3.2. Lesser Wings

The LWs of the SB project horizontally from the anterosuperior aspect of the sphenoid body, forming a clear boundary between the anterior and middle cranial fossae. Each wing supports the roof of the orbit and ends medially at the ACP, which serves as an attachment point for the tentorium cerebelli [5]. Between the LWs and GWs is the superior orbital fissure, an oblique opening that functions as an essential passage for cranial nerves III (oculomotor), IV (trochlear), V1 (ophthalmic division of the trigeminal), and VI (abducens), along with the superior and inferior ophthalmic veins [5].

### 3.3. Greater Wings

The GWs of the SB extend laterally and posteriorly from the central body and are divided into distinct surfaces—cerebral, orbital, temporal, infratemporal, and maxillary—based on their anatomical relationships with nearby regions [5]. These surfaces play a role in forming important cranial structures, including the floor of the middle cranial fossa, the lateral wall of the skull, and the orbit. Within the GWs are several key foramina that act as pathways for neurovascular structures [5] (Figure 2):

- FR: transmits the maxillary nerve (V2).
- FO: transmits the mandibular nerve (V3) along with accessory meningeal vessels.
- FS: transmits the middle meningeal artery and vein, and the meningeal branch of the MN (V3).

Posterior to the FS lies the spine of the sphenoid—a pointed bony projection that serves as an attachment site for the SML, whose embryological development has been previously described [20].

### 3.4. Pterygoid Processes

Extending inferiorly from the junction of the SB and GW, each PP process consists of two plates: a medial and a lateral plate. The medial pterygoid plate (MPP) tapers into a hook-like projection known as the pterygoid hamulus, which serves as a pulley for the tendon of the tensor veli palatini muscle [5]. The space between the MPP and LPP forms the pterygoid fossa, which provides an origin site for portions of the medial pterygoid muscle [5]. A key anatomical feature of this region is the pterygoid canal, also known as the Vidian canal, which traverses the base of the pterygoid process. It transmits the nerve of the pterygoid canal (Vidian nerve) and accompanying artery, linking the foramen lacerum (FL) to the pterygopalatine fossa [5].

## 4. Anatomical Variations of the Human Adult Sphenoid Bone

The adult human SB shows significant morphological variability. These differences include variations in ossification patterns, the presence of accessory foramina or canals, and structural anomalies of bone and ligament components. Such variants may be congenital, often reflecting deviations in embryological development, or may develop as age-related changes acquired over time [24].

### 4.1. Sella Anatomy—Clinoid Process Variations and Sellar Bridges

The sella is a saddle-shaped depression in the body of the SB that houses the pituitary gland [25]. It is bordered by three pairs of clinoid processes—ACP, MCP, and PCP—which can vary in shape. These variations often appear as bony connections between the processes, known as sellar or interclinoid bridges (ICBs) [26,27]. These bridges can have significant clinical implications because of their proximity to vital neurovascular structures. Iskra et al. [25] performed a systematic review and meta-analysis involving 18,364 patients, reporting a pooled average sella depth of 6.59 mm and a mean volume of 845.80 mm^3^. Normal sella morphology without bridging was observed in 55.56% of cases. Although the study did not examine geographic or methodological influences, these measurements provide baseline values for assessing sellar conditions such as hypopituitarism, adenomas, and empty sella syndrome [25]. Roomaney and Chetty [28] noted atypical sella shapes—such as J-shaped, deep, or flattened dorsum sellae—in individuals with craniofacial syndromes, including Down syndrome, Williams syndrome, and Axenfeld–Rieger syndrome. They linked these anomalies to disrupted sphenoid ossification and neural crest migration, supported by embryological models from Kjaer et al. [29].

The ACP, an extension of the LW, shows significant variation with both developmental and surgical importance. Its morphology can impact nearby neurovascular structures and affect surgical access to the parasellar region. In a CT-based study, Mikami et al. [30] found pneumatization of the ACP in 9.2% of individuals. Most pneumatizations originated from the sphenoid sinus (81.8%), with some extending from the ethmoid sinus (10.9%) or both (7.3%). These were classified into three types based on their route: Type I (via the optic strut, 74.5%), Type II (via the anterior root, 14.5%), and Type III (via both, 10.9%) [30]. Rennert et al. [31] demonstrated that the ACP undergoes three phases of elongation during development, increasing in length by 49% from infancy to adulthood. ACP pneumatization was not present before age 11, while ossified caroticoclinoid and interclinoid ligaments appeared in 15% of subjects as young as 3 years [31].

The MCP is a variable projection of the sphenoid bone that can be frequently absent. Sharma et al. [32] conducted a large morphometric study of 2726 skulls and identified clinically relevant middle clinoid processes in 42% of specimens. They classified middle clinoid processes into three categories:

- Incomplete (69% of all middle clinoid processes): bony projections that do not reach the anterior clinoid process.
- Contact (4%): middle clinoid processes that contact the anterior clinoid process without forming a true ring.
- Caroticoclinoid Ring (27%): complete ossification forming a bony ring around the internal carotid artery.

Key morphometric data were also recorded as the middle clinoid processes were predominantly located in the anterior one-third of the lateral bone window. Middle clinoid processes were more prevalent in white individuals and with increasing age, suggesting ossification may progress with time. A statistically significant association was noted between caroticoclinoid foramen formation and interclinoid bridges [32].

The PCP is probably the less frequent studied structure with only few variations described, mainly the interclinoid bridges. Nevertheless, Salma et al. [33] reported on the variations in posterior clinoid processes based on 100 dry human skulls. The posterior clinoid process was classified as normal (78%), elongated (16%) and pneumatized (6%) [33].

**Figure 3:**
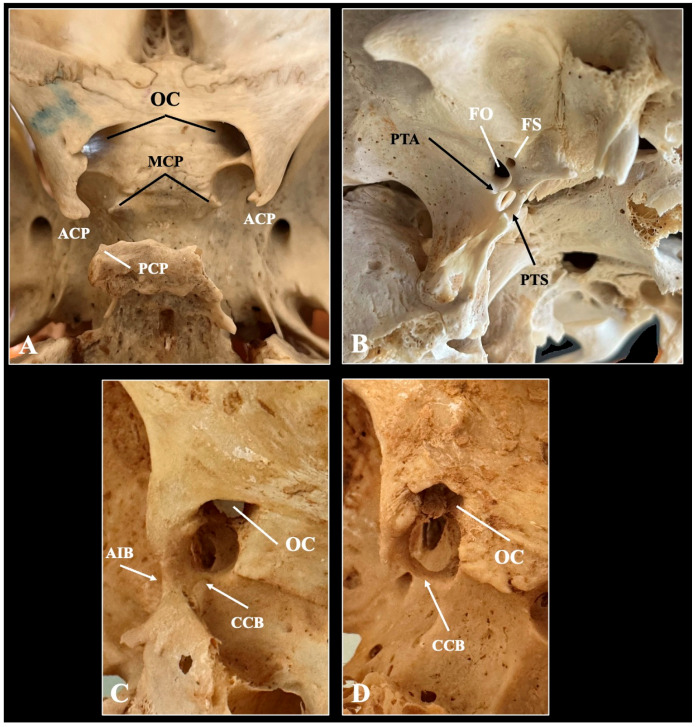
Typical (**A**) and variant anatomy (**B**–**D**) of the sella region depicted through osteological specimen. ACP—anterior clinoid process, MCP—middle clinoid process, PCP—posterior clinoid process, OC—optic canal, PTA—pteryogoalar bar, PTS—pterygospinous bar, FO—foramen ovale, FS—foramen spinosum, CCB—caroticoclinoid bar, AIB—anterior interclinoid bar.

Sellar bridges are osseous connections between the clinoid processes of the sphenoid bone and represent important anatomical variants of the sellar region. They are typically formed through the ossification of intracranial ligaments and include the caroticoclinoid bridge (CCB) and interclinoid bridge (ICB) [24]. These bridges give rise to additional foramina that can influence both the surrounding neurovascular structures and neurosurgical approaches to the skull base. Sellar bridges are most frequently categorized according to the clinoid processes they connect based on Nikolova et al. [26] (Figure 3):

- Type I (caroticoclinoid bridge): Connects the anterior and middle clinoid processes, formatting the CCF, also known as the anterior interclinoid canal. This foramen transmits the ICA.
- Type II (interclinoid bridge between anterior, middle, and posterior clinoid processes): forms a CCF anteriorly and a posterior interclinoid foramen posteriorly.
- Type III (Interclinoid bridge between anterior and posterior clinoid processes): encircles a common interclinoid foramen (canal of Gruber), transmitting both the ICA and venous structures.
- Type IV (Bridge between middle and posterior clinoid processes): rarely observed and typically not classified separately in most anatomical studies.

The CCF is an anatomical variation formed by ossification of the caroticoclinoid ligament that extends between the ACP and MCP [33] (Figure 3). This ossified bridge results in a foramen through which the ICA pass [34]. Although considered a variant, the CCF is not uncommon. Zdilla et al. [16] explored the fetal development and observed that accessory ossification centers in the presphenoid region may contribute to asymmetrical development of the clinoid processes. This early variation potentially sets the stage for CCF formation in postnatal life. The ossification of the ligament typically begins in early childhood, though the exact timeline varies. Developmental persistence of cartilaginous or ligamentous structures into bone leads to the formation of the CCF and other sellar bridges [15]. During our recent meta-analysis, we estimated the pooled prevalence of the caroticoclinoid ligament ossification in 17.47% of the population [34]. The pooled mean diameter of the CCF was estimated at 5.00 mm [34]. The nationality, type of study, side, and sexes did not influence the estimated pooled prevalence on the meta-analysis [34]. The variation can be accurately detected through CT-imaging [35], while we previously proposed three morphological compression patterns to the internal carotid artery. Specifically, when the CCF has a diameter less than 4.0 mm, it was considered to pose a high risk of compression to the internal carotid artery and was identified in 16.8% of the sample with a female predominance [35].

The ICB is less well understood. Natsis et al. [26] reported a prevalence of 17.57% for sellar bridges in 148 Greek skulls, with 61.5% of these being bilateral. Type I (CCF formation) was the most common variant. Morphometric data indicated that the formation of bridges decreased the distance between the clinoid processes, potentially narrowing surgical pathways [26]. In a CT study of 315 Bulgarian individuals, Nikolova et al. [27] found sellar bridges in 21.7% of cases—Type I was again the most common (14.3%), followed by Types II and III (7.4%), with no Type IV bridges observed. There were no significant differences in sex or laterality [27]. Serioli et al. [36] reported a co-occurrence of complete CCF and ICB in 4% of CT scans and up to 6.3% of anatomical specimens. The presence of multiple bridges may increase surgical risks near the cavernous sinus, emphasizing the importance of tailored imaging and surgical planning [36].

### 4.2. Variations of the Typical Sphenoid Foramina

The FO was classically described as oval in shape and singular in structure; however, modern anatomical studies reveal a wide spectrum of morphological variability in its shape, dimensions, orientation, and even patency [37]. The shape of the FO has been described using numerous terms including oval, elongated, semicircular, almond, D-shaped, pear-shaped, slit-like, and irregular [37]. These descriptors, however, are often subjective and lack consistency across studies. To provide more objective characterization, Zdilla et al. [37] applied geometric and morphometric indices such as circularity, solidity, roundness, and aspect ratios, showing that the FO often deviates from being perfectly oval. Bony spurs, tubercles, and septations may partially divide the FO, leading to multilobed or duplicated foramina [37]. Some foramina were irregularly contoured or segmented by thin bony spicules, occasionally resulting in two or three distinct openings within the same osseous niche [37]. Additionally, cases of FO absence or confluence with the FS have rarely been reported [37]. Furthermore, Triantafyllou et al. [38] proposed a novel evidence-based classification system based on morphometric measurements. Thus, they described three patterns based on the relationship of the anteroposterior and lateromedial distances [38]. Iwanaga et al. [39] conducted a detailed anatomical study to classify the positional relationship between the FO and the base of the lateral pterygoid plate—an important surgical landmark during percutaneous treatments for trigeminal neuralgia. Based on their analysis of 160 sides from 80 skulls, four types of relationships were defined:

- Type I (lateral)—FO located medial to the posterior border of the lateral pterygoid plate (29%).
- Type II (medial)—FO located lateral to the posterior border (15%).
- Type III (direct)—FO aligned directly with the posterior border (35%).
- Type IV (removed)—FO disconnected from the lateral pterygoid plate, making surgical access more difficult (21%).

Triantafyllou et al. [38] identified similar types and observed that the ossified PTAL and PTSL alter this relationship.

The FS, located posterolateral to the FO on the greater wing of the sphenoid bone, exhibits notable morphological variation in shape, size, and frequency of presence. It serves as a critical conduit for the middle meningeal artery and vein and the meningeal branch of the V3. Bhattarai et al. [40] analyzed FS morphology via CT and reported a mean length and width of 2.38 ± 0.36 mm and 1.94 ± 0.30 mm, respectively, with an average area of 3.69 ± 0.95 mm^2^. They found statistically significant sex-based differences, with male skulls consistently presenting larger FS dimensions than female ones. However, age and lateral asymmetry showed no significant association. Sugano et al. [41] highlighted developmental absence or hypoplasia of FS, particularly in cases with anomalies in the middle meningeal artery, where FS may be absent, duplicated, or display atypical emergence patterns.

The FR, located anteromedial to the FO, transmits the V2 and is classically described as a round, symmetric opening. However, it also exhibits anatomical variability in size, orientation, and even duplication. Bhattarai et al. [40] provided mean morphometric measurements for FR: a height of 2.41 ± 0.49 mm, width of 2.40 ± 0.55 mm, and area of 4.58 ± 1.49 mm^2^. Like the FS, the FR also showed sex-based differences in dimensions but no significant side asymmetry [40]. Rusu [42] described a rare case of duplicated foramen rotundum, associated with a fenestrated maxillary nerve, during cadaveric dissection. This anomaly involved an osseous lamella separating two canals, each transmitting part of a split V2 nerve, which later reunited in the pterygopalatine fossa. Triantafyllou et al. [43] and Syed et al. [44] also reported the duplicated FR through osteological observation. Inal et al. [45] found strong correlations between the FR and surrounding structures, including the optic canal (OC) and Vidian canal (VC). Their study revealed that the width and area of the FR were positively correlated with paranasal sinus pneumatization, suggesting that sinus development may indirectly influence FR morphology and position [45].

The VC, the so-called pterygoid canal, traverses the sphenoid bone from the foramen lacerum to the pterygopalatine fossa. Its morphology and relationship to surrounding structures exhibit notable anatomical variation. Several studies have established classification systems based on the relationship between the VC and the sphenoid sinus. Yegin et al. [46] described: Type 1- VC entirely within the sphenoid sinus (most common), Type 2—partially protruding into the sinus, Type 3—entirely embedded in the sphenoid bone (least exposed). Additionally, based on the sphenoid floor, they described four types (Types 1–4), depending on whether the VC lies at, above, or below the sinus floor, or within a V-shaped depression. In their study of 594 patients using CT imaging, they found that type 1 was the most prevalent VC type (~33–36%). Bony dehiscence (absence of a complete bony roof) of the VC was observed in 22.2–26.6% of cases, more often on the left side [46].

The OC, located within the lesser wing of the sphenoid, transmits the optic nerve (CN II) and the ophthalmic artery. Although classically described as a singular canal with predictable morphology, several anatomical variations have been documented, particularly involving accessory canals, duplication, and altered canal trajectories. One of the rare but clinically significant variants is the accessory optic canal (AOC)—also referred to as a duplicated optic canal or ophthalmic canal [47]. In this configuration, a second canal, typically inferolateral to the main OC, houses the ophthalmic artery while the main canal transmits the optic nerve. Zdilla et al. [47] observed that the presence of an AOC is associated with a smaller main optic canal in terms of area, perimeter, and minor axis length. Triantafyllou et al. [48] described a rare bilateral variant featuring well-defined accessory canals inferolateral to each OC in an adult skull. These accessory structures, likely transmitting the ophthalmic artery, highlight the necessity for preoperative imaging awareness, particularly in orbital and skull base surgery. Ugradar et al. [49] provided additional insight into OC entry point variability into the orbit, describing two main types of canal trajectories based on CT analysis. In 90% of cases (Type 1), the optic canal coursed through the lateral wall of the sphenoid sinus, entering the orbit medially. In 10% (Type 2), the canal followed a superior trajectory through the sphenoid sinus roof [49]. The entry angle differed significantly, with implications for surgical access and risk of optic nerve damage during endoscopic or transcranial approaches [49].

The superior orbital fissure (SOF), a vital anatomical cleft between the greater and lesser wings of the sphenoid bone, serves as a conduit for several cranial nerves (III, IV, V1, VI) and vessels connecting the orbit with the middle cranial fossa. Its morphology, dimensions, and compartmental organization are highly variable, with implications for neurosurgical and ophthalmologic procedures. Natori and Rhoton [50] provided a foundational anatomical classification of the SOF into three distinct sectors based on its neurovascular content:

- The lateral sector transmits the trochlear, frontal, and lacrimal nerves along with the superior ophthalmic vein—all outside the annular tendon.
- The central sector (oculomotor foramen) contains the superior and inferior divisions of the oculomotor nerve, abducens nerve, nasociliary nerve, and sympathetic roots, all passing through the annular tendon.
- The inferior sector transmits the inferior ophthalmic vein beneath the annular tendon and is filled with posterior orbital fat.

These divisions reflect the complexity of neurovascular relationships within the fissure and highlight the importance of its spatial configuration during surgical approaches to the cavernous sinus and orbital apex. In a modern morphometric study, Pirinc et al. [51] used three-dimensional reconstruction of computed tomography imaging to assess 400 SOFs in 200 individuals. They proposed a novel classification of SOF shapes based on reference lines dividing the fissure into upper and lower openings. Seven morphotypes were described: curved, straight, racket-shaped (most common), eight-shaped, keyhole, narrow (least common), and triangular. This classification offers an objective framework for understanding the diversity in SOF morphology. Notably, right-sided SOFs were significantly wider in females, while no sex-based differences were found in length [51]. Govsa et al. [52] further emphasized SOF variability in cadaveric dissections, confirming the high frequency of asymmetry between sides and variable bony bridges or spurs that may narrow or compartmentalize the fissure [52].

### 4.3. Presence of Emissary Foramina

Emissary foramina of the sphenoid bone represent important anatomical variants through which emissary veins connect intracranial venous sinuses with extracranial venous plexuses. The sphenoidal emissary foramen (SEF), also known as the foramen of Vesalius, is located anteromedial to the foramen ovale and transmits an emissary vein from the pterygoid venous plexus to the cavernous sinus [53]. Its prevalence is highly variable across studies and populations. On their meta-analysis, Piagkou et al. [53] estimated the pooled prevalence of the SEF in approximately 33% of the population. Leonel et al. [54] highlighted the morphological variability of the SEF, observing it as either round or oval and ranging in diameter from 0.5 mm to 2.5 mm. The SEF may coexist with ossified ligaments (PTAL or PTSL), which can complicate percutaneous access to the FO [38]. Triantafyllou et al. [38] identified that while the SEF does not significantly alter the relationship between the FO and the lateral pterygoid plate, its presence should be acknowledged in surgical planning due to its potential venous connections. A much rarer variant, the accessory sphenoidal foramen, has been reported by Triantafyllou et al. [55] in 0.6% of skulls. This foramen is found in the middle cranial fossa, posterior to the FR and anterior to the FO. It was observed to open into the infratemporal fossa and was hypothesized to transmit accessory vascular structures such as the pterygomeningeal artery or emissary veins. Accessory sphenoidal foramen may coexist with one or more SEFs, as reported in the same study, highlighting the complexity and overlapping venous architecture of the sphenoid region [55]. Lastly, in 2024, Rusu (2024) first described a newly emissary pathway, the sphenopterygoid canal (SPC). Rusu [56] and Triantafyllou et al. [43] described this structure as a variable, elongated canal often found anterolateral to the FO and near the lateral pterygoid process. Its contents likely include emissary veins [56].

### 4.4. Variations of the Ligaments

The SB is associated with several ligaments that may undergo partial or complete ossification, producing clinically significant bony structures. The most relevant among these are the PTAL, PTSL, and the SML [57].

The PTAL, extending from the root of the lateral pterygoid plate to the undersurface of the greater wing of the sphenoid, can ossify and form a PTA bar. During their meta-analysis, Pekala et al. [58] estimated the pooled prevalence of the complete PTAL ossification in 4.4% and the incomplete ossification in 8.4%. Tubbs et al. [59] found PTAL ossification in 2.6% of 154 skulls examined, with complete bony foramen formation (foramen of Hyrtl) observed in 1.3%. Moreover, Triantafyllou et al. [38] confirmed that PTAL ossification significantly alters the spatial relationship between the FO and the lateral pterygoid plate. Matys et al. [60] noted that PTAL ossification is more common in males and predominantly occurs unilaterally. Its presence may compress branches of the MN, potentially contributing to neuralgia or atypical facial pain [60].

The PTSL extends from the posterior border of the lateral pterygoid plate to the spine of the sphenoid. When ossified, it forms the PTS bar (Civinini’s ligament), creating the foramen of Civinini. Henry et al. [61] performed a systematic review with meta-analysis of 35 studies and 14,047 subjects, and they identified the complete PTSL ossification in 4.4% and the incomplete ossification in 11.6%. Tubbs et al. [59] reported complete PTSL ossification in 1.3% of skulls and incomplete ossification in an additional 2.6%. Cho et al. [9] described the developmental origins of the PTSL in fetal and pediatric skulls, indicating that early cartilaginous connections between the lateral pterygoid and sphenoid spine may persist and ossify. Esen et al. [62] found that the ossification frequency increases with age, supporting a gradual ossification process that may reflect biomechanical stress or genetic predisposition.

The SML, a remnant of Meckel’s cartilage, extends from the spine of the sphenoid to the lingula of the mandible. While usually a flat, fibrous band, anatomical variants have been observed. Simonds et al. [63] reported a rare duplication of the SML, with dual origins from both the sphenoid spine and the mandibular fossa of the temporal bone. Unlike the PTAL and PTSL, the SML does not ossify into a bar-like structure [63].

## 5. Clinical and Surgical Associations of the Human Adult Sphenoid Bone

The SB’s central location at the skull base and its involvement in multiple neurovascular pathways make it a key focus in clinical and surgical practice. Morphological variations, including changes in foraminal shape, emissary venous routes, and ossified ligaments, can complicate surgical approaches or completely restrict access to the surgical site. These variations present challenges during neurosurgical, endoscopic, and maxillofacial procedures. Accurate preoperative identification through advanced imaging is crucial for minimizing iatrogenic risks and enhancing surgical accuracy and patient outcomes.

### 5.1. Neurosurgical Considerations: Sellar and Parasellar Region

The sellar region harbors the pituitary gland and is surrounded by critical structures such as the ICA, cavernous sinuses, and optic structures [5]. Variants like CCF and ICBs may result in bony rings encasing the ICA, leading to potential compression or altered hemodynamics [34]. Surgical approaches to pituitary adenomas or parasellar tumors must account for such bridges, as their presence may narrow operative corridors or increase risk during anterior clinoidectomy [35]. Small-diameter CCF (<4.0 mm) have been identified as high-risk configurations for ICA compression, especially in females [35]. Furthermore, ICBs—especially Type II and III configurations—may obscure direct transsphenoidal access or require modification of standard endoscopic routes, such as anterior clinoidectomy procedures [26].

### 5.2. Cranial Nerves’ Compression from Sphenoid Bone and Its Variations

Percutaneous procedures for trigeminal neuralgia rely on accurate cannulation of the FO [39]. However, the presence of ossified PTALs or PTSLs can impede needle access, leading to procedural failure or neurovascular injury [59]. The PTAL and PTSL, when ossified, may obscure the FO or distort its anatomical orientation (topography) relative to the LPP [39]. Additionally, EF, such as SEF, may transmit EVs that connect the pterygoid plexus to the cavernous sinus. These pose a risk of cavernous sinus thrombosis or hemorrhage if inadvertently damaged during trigeminal blocks or percutaneous rhizotomies [53,56].

### 5.3. Endoscopic Skull Base Surgery and Orbital Approaches

Modern endonasal endoscopic approaches require precise navigation through the sphenoid sinus and nearby foramina (e.g., pterygoid canal, OC, and SOF). Variations in pterygoid canal anatomy, especially dehiscence or altered paths [46], can complicate approaches to the petrous ICA [64], Meckel’s cave [65], or the pterygopalatine fossa [66]. AOC paths increase the risk of damaging the optic nerve or ophthalmic artery (ON or OA) during decompression or tumor removal [47,49]. Similarly, SOFs may have bony spurs or divisions, altering the orientation of cranial nerves III, IV, V1, and VI, making careful preoperative imaging essential to prevent postoperative diplopia or ophthalmoplegia [52].

### 5.4. Oral and Maxillofacial Surgery

SML and its attachment to the sphenoid’s spine may affect the position of the inferior alveolar nerve, potentially complicating V2 anesthesia. Anomalous attachments or duplications, as reported by Simonds et al. [63], can lead to failed anesthesia or iatrogenic nerve injury during procedures such as mandibular osteotomies or third molar extractions [67]. Additionally, altered ossification of the sphenoid may cause unexpected foramina variants, like duplicated FR [42,43], which could mislead surgical orientation or complicate regional anesthesia targeting the maxillary nerve (V2).

### 5.5. Radiological and Diagnostic Implications

High-resolution CT and MR imaging have become indispensable for identifying sphenoidal variants preoperatively. Studies highlight the importance of recognizing sellar bridges, foraminal anomalies, and ossified ligaments in neuroimaging to prevent misdiagnoses, such as mislabeling ossified structures as fractures or tumors [34,35]. The increased use of imaging techniques allows researchers to identify even previously undescribed foramina of the human skull [56].

## 6. Conclusions

The SB is a complex and developmentally dynamic part of the human skull base. Its embryological development involves a coordinated sequence of endochondral and intramembranous ossification from multiple cartilaginous centers, which undergo spatial remodeling in response to neural, vascular, and biomechanical cues around them. These early processes create the mature sphenoid’s intricate shape—featuring many foramina, canals, and ligament attachments—all of which can vary significantly. In adults, the SB plays vital roles in the cranial base, orbit, and various fossae, acting as a pathway for important cranial nerves and blood vessels. Variants, such as ossified ligaments (PTAL and PTSL), unusual foramina (e.g., duplicated FR or pathways like SEF and SPC), and sellar bridges (including CCF, ICB), are common and hold clinical significance. These variants are linked to neurovascular compression syndromes, changes in blood flow dynamics, and higher surgical risk during skull base procedures. Understanding the sphenoid’s developmental biology, standard anatomy, and variant forms is essential for clinicians and surgeons working in this region. Advances in high-resolution CT, 3D reconstruction, and morphometric modeling have enhanced the detection of subtle anatomical anomalies prior to surgery. Combined with the expansion of anatomical databases and meta-analyses, these technologies continue to refine preoperative planning, reduce complications, and enhance patient outcomes in neurosurgery, maxillofacial procedures, and endoscopy.

## Figures and Tables

**Table 1:** Timeline of ossification centers in sphenoid bone development. This table synthesizes findings from multiple sources [3,6,10,11,14] and outlines the developmental trajectory of key sphenoidal components. LWs = lesser wings; GWs = greater wings; PPs = pterygoid processes; PB = posterior body; AB = anterior body.

Ossification Center	Anatomical Region	Type	Approximate Onset	Notes
Basisphenoid	PB	Primary	40–45 days	First visible ossification center
Presphenoid	AB	Primary	16 weeks	Fuses later with basisphenoid
Orbitosphenoid	LWs	Secondary	8–9 weeks	Derived from membranous ossification
Alisphenoid	GWs	Mixed	8–9 weeks	Includes both cartilage and membrane
Medial and lateral pterygoid plates	PPs	Secondary	12–14 weeks	Ossify from independent centers
Intrasphenoidal synchondrosis	Body (midline)	Synchondrosis	Fuses postnatally	Key for skull base elongation
Sphenoethmoidal synchondrosis	Anterior base	Synchondrosis	Late fetal	Fuses by 7–8 years

## Data Availability

All the data are available upon reasonable request to the corresponding author.

## Abbreviations

ACP	Anterior Clinoid Process.
AOC	Accessory Optic Canal.
CCF	Caroticoclinoid Foramen.
CCB	Caroticoclinoid Bar.
CN	Cranial Nerve.
CRL	Crown–Rump Length.
CT	Computed Tomography.
EV	Emissary Vein.
FO	Foramen Ovale.
FR	Foramen Rotundum.
FS	Foramen Spinosum.
GWs	Greater Wings (of the sphenoid).
ICA	Internal Carotid Artery.
ICB	Interclinoid Bridge.
LPP	Lateral Pterygoid Plate.
LWs	Lesser Wings (of the sphenoid).
MCP	Middle Clinoid Process.
MN	Mandibular Nerve.
MPP	Medial Pterygoid Plate.
OA	Ophthalmic Artery.
OC	Optic Canal.
ON	Optic Nerve.
PPs	Pterygoid Processes.
PCP	Posterior Clinoid Process.
PTSB	Pterygospinous Bar.
PTSL	Pterygospinous Ligament.
PTAL	Pterygoalar Ligament.
SB	Sphenoid Bone.
SEF	Sphenoidal Emissary Foramen.
SML	Sphenomandibular Ligament.
SOF	Superior Orbital Fissure.
SPC	Sphenopterygoid Canal.
VC	Vidian Canal (Pterygoid Canal).
V2	Maxillary Division of Trigeminal Nerve.
V3	Mandibular Division of Trigeminal Nerve.
